# Screening and Spontaneous Mutation of Pickle-Derived *Lactobacillus plantarum* with Overproduction of Riboflavin, Related Mechanism, and Food Application

**DOI:** 10.3390/foods9010088

**Published:** 2020-01-14

**Authors:** Ying-Ying Ge, Jia-Rong Zhang, Harold Corke, Ren-You Gan

**Affiliations:** 1 Department of Food Science & Technology, School of Agriculture and Biology, Shanghai Jiao Tong University, Shanghai 200240, China; gy1994@sjtu.edu.cn (Y.-Y.G.); zhangjiarong@sjtu.edu.cn (J.-R.Z.); hcorke@sjtu.edu.cn (H.C.); 2 Research Center for Plants and Human Health, Institute of Urban Agriculture, Chinese Academy of Agricultural Sciences, Chengdu 610213, China

**Keywords:** vitamin B2, *Lactobacillus*, pickle juice, roseoflavin mutation, fermented soymilk

## Abstract

Riboflavin, also known as vitamin B2, plays an important role in human cell metabolism and participates in various redox reactions and in energy utilization. In this study, 90 riboflavin-producing lactic acid bacteria (LAB) were screened out from pickle juices. The yields of riboflavin in these LAB were about 0.096–0.700 mg/L, and one strain, *Lactobacillus plantarum* RYG-YYG-9049, was found to produce the highest riboflavin content. Next, roseoflavin was used to induce the spontaneous mutation of RYG-YYG-9049, and selected roseoflavin-resistant colonies generally produced higher riboflavin contents, ranging from 1.013 to 2.332 mg/L. The No. 10 mutant, *L. plantarum* RYG-YYG-9049-M10, had the highest riboflavin content. Next, the molecular mechanism of enhancing riboflavin production in RYG-YYG-9049-M10 was explored, leading to the finding that roseoflavin treatment did not change the *rib* operons including the *ribA*, *ribB*, *ribC*, *ribH*, and *ribG* genes. Unexpectedly, however, this mechanism did induce an insertion of a 1059-bp DNA fragment in the upstream regulatory region of the *rib* operon, as compared to the wild-type RYG-YYG-9049. To the best of our knowledge, this is the first report that roseoflavin could induce an insertion of DNA fragment in LAB to increase riboflavin content, representing a new mutation type that is induced by roseoflavin. Finally, in order to fortify riboflavin content in soymilk, RYG-YYG-9049 and RYG-YYG-9049-M10 were used to ferment soymilk, and several fermentation parameters were optimized to obtain the fermented soymilk with riboflavin contents of up to 2.920 mg/L. In general, roseoflavin induction is an economical and feasible biotechnological strategy to induce riboflavin-overproducing LAB, and this strategy can be used to develop LAB-fermented functional foods that are rich in riboflavin.

## 1. Introduction

Riboflavin, known as vitamin B_2_, is an important vitamin for humans and has been selected as one of the six major indicators for assessing human growth, development, and nutritional status by the World Health Organization (WHO) [1]. About 0.3–1.8 mg of riboflavin per day is required for an adult to keep healthy [1]. However, riboflavin cannot be synthesized in the human body and must be obtained from the diet [2,3]. It has also been reported that riboflavin has the ability to form complexes with certain metal ions, and its supplementation may increase the cellular uptake of zinc and iron [4]. Thus, riboflavin has direct and indirect effects on human growth and development. Additionally, riboflavin deficiency or the defective transport of riboflavin can cause neurological disorders, cataracts, cardiovascular abnormalities, and even various cancers [5,6]. To date, riboflavin deficiency is still a common and serious health concern in both developing and developed countries [7].

**Figure 1:**
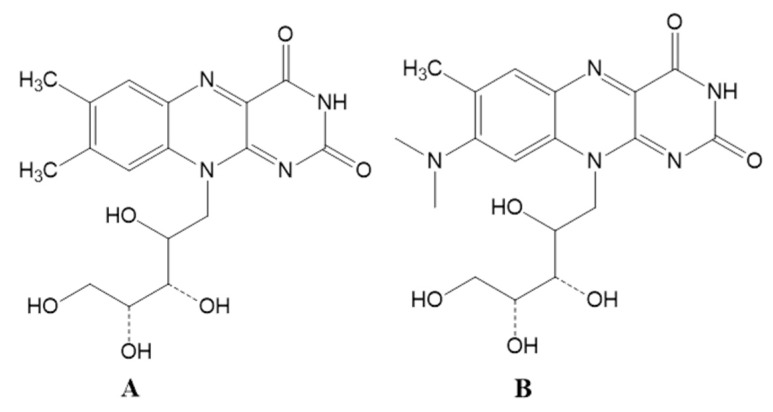
The chemical structures of riboflavin (**A**) and its analog roseoflavin (**B**).

At present, riboflavin is mainly synthesized in the industry via microbial biosynthesis, and the commonly used flavinogenic microorganisms are fungi and bacteria. The main industrial producer strains of riboflavin include two yeast-like fungi *Eremothecium ashbyii* and *Ashbya gossypii*, as well as the bacteria *Bacillus subtilis* [8]. Compared to fungal fermentation, bacterial fermentation has several advantages for riboflavin biosynthesis, such as a short fermentation time, simple culture media, and the mature genetic engineering technologies applied to prokaryotic bacteria [9,10]. Lactic acid bacteria (LAB) are a group of probiotic bacteria that are closely related to human life, and they have been widely used in the food industry to expand the source and diversity of food, as well as to improve the nutritional value of food. It has been found that certain LAB with complete riboflavin operons, including the *ribA*, *ribB*, *ribH*, and *ribG* genes, possess the ability to biosynthesize riboflavin [11]. Roseoflavin (Figure 1), the toxic analog of riboflavin, can cause the spontaneous mutation of LAB, mainly by altering the gene regulatory region of the riboflavin operon, and, interestingly, most mutant LAB strains exhibit enhanced riboflavin production. Therefore, roseoflavin is often used to induce the spontaneous mutation of LAB in order to obtain the LAB strains with high riboflavin productivity [12].

Soybean is a popular food that is rich in high-quality protein and dietary polyphenols, especially isoflavones [13,14,15], and its commercial products have been steadily increasing [16]. Soymilk is a popular beverage in many countries. Because of its absence of cholesterol, gluten, and lactose, soymilk is also suitable for people with lactose intolerance, vegetarians, and milk-allergic patients [17]. However, soybeans contain a low level of riboflavin that is even lower in soymilk due to its loss in the processing stages [18].

It has been reported that the content of riboflavin in soymilk can be markedly enhanced by fermentation with riboflavin-overproducing LAB strains [19]. Therefore, this study was conducted to discover new LAB strains with high riboflavin productivity via natural screening and roseoflavin-induced spontaneous mutation, as well as to investigate related mechanisms of roseoflavin-mediated riboflavin overproduction in selected LAB mutant strains, which were finally applied for the development of fermented soymilk that is rich in riboflavin. The key issue was to find the mutation site of the *Lactobacillus* mutant in order to identify the reason for high riboflavin production in the mutant strain.

## 2. Materials and Methods

### 2.1. Chemicals and Reagents

The de Man-Rogosa-Sharpe (MRS) broth was purchased from OXOID (Hants, UK). A chemically defined medium (CDM) was purchased from Becton, Dickinson and Company (Sparks, MD, USA). A TIANamp Bacteria DNA Kit was purchased from Tiangen Biotech Company (Beijing, China). Methanol (HPLC grade) was purchased from Amethyst Chemicals (Beijing, China). Formic acid and acetic acid were purchased from Shanghai Ling Feng Chemical Reagent Company (Shanghai, China). Deionized ultrapure water was used in all the experiments.

### 2.2. Isolation of the Riboflavin-Producing LAB Strains from Pickle Juices

Eleven commercial pickles (Shanghai Yige Food Trading Co., Ltd., Shanghai, China) were bought from a local supermarket (Auchan, Shanghai, China), and all the pickle juices were aseptically collected and mixed. The pickle juice mixture was diluted with a sterile phosphate buffer solution (PBS) (1:1000, *v*/*v*), and then 100 μL was plated onto a plate coated with a CDM that was adapted by the removal of riboflavin [20]. The riboflavin assay was performed according to the instructions provided by the manufacturer (BD Difco, Sparks, MD, USA). The plate was placed in an anaerobic jar and incubated at 37 °C for 48 h. At the end of the incubation process, 90 visible complete LAB colonies were selected on the CDM agar plate and subsequently cultured in a liquid CDM at 37 °C for 24 h before being reactivated three times. All bacteria were finally stored at −80 °C in an MRS broth supplemented with 25% glycerol.

### 2.3. Extraction of Riboflavin

The extraction of riboflavin in the culture media and in the bacterial cells was carried out by using the method that was previously described by Juarez del Valle [19], with minor modifications. 500 μL of the culture media was double diluted with a 1% (*v*/*v*) acetic acid aqueous solution and mixed by vortex. The sample was next centrifuged at 21,400× *g* for 5 min at 4 °C, and the supernatant was collected. The cell pellet was resuspended in 500 μL of a 1% (*v*/*v*) acetic acid aqueous solution. After heating at 95 °C for 6 min, the mixture was centrifuged at 21,400× *g* for 5 min at 4 °C, and the supernatant was collected. Finally, two supernatants were combined and stored at −20 °C for the further quantification of riboflavin.

### 2.4. Quantification of Riboflavin Content by HPLC

The concentration of riboflavin was determined by high-performance liquid chromatography (HPLC) coupled with a photodiode array (PDA) detector (Shimadzu, Kyoto, Japan) based on a previous study [19]. A Shim-pack GIS C18 column (4.6 × 250 mm, 5 μm) (Shimadzu, Kyoto, Japan) was used to separate riboflavin in the culture media or the LAB-fermented soymilk. The sample was passed through a 0.22 μm pore size membrane filter. The mobile phase consisting of methanol/water/formic acid (70:29.25:0.75, *v*/*v*/*v*) was used to elute riboflavin. All solvents used in the mobile phase were HPLC-grade and degassed with ultrasound for 30 min. The flow speed was 0.8 mL/min, the injection volume was 20 μL, and the detection wavelength was set at 267 nm. A standard curve was prepared based on a riboflavin standard (concentration range 0.2–5 mg/L, R^2^ = 0.9996). By comparing the retention time and peak area of the standard sample, the content of the sample was determined.

### 2.5. Identification of LAB Species

The high riboflavin-producing LAB were separated and purified three times in MRS agar plates, and the obtained strain was finally cultured in MRS media and examined via Gram staining and a catalase test [21,22]. The genomic DNA of the strain was extracted by using a TIANamp Bacteria DNA Kit, and 16S rDNA was sequenced (Beyotime Biotechnology, Shanghai, China). According to the method of Capozzi et al. [23], the 16S rDNA was amplified by PCR with universal primers 27F (5′-AGAGTTTGATCCTGGCTCAG-3′) and 1492R (5′-AAGGAGGTGATCCAGCCGCA-3′). DNA sequencing data were subjected to the database of National Center for Biotechnology Information (NCBI) Basic Local Alignment Search Tool (BLAST) for homology comparison, and a similarity higher than 98% to 16S rDNA sequences of known strains was used as the criteria for species identification [24].

### 2.6. Inducing LAB Mutation by Roseoflavin

The RYG-YYG-9049 strain with the highest riboflavin production was selected for inducing LAB mutation, and the procedure was performed based on a previous study with some modifications [25]. RYG-YYG-9049, at the logarithmic growth phase, was inoculated into a CDM agar plate that contained 100 mg/L of roseoflavin and anaerobically incubated for 2–4 days at 37 °C. Single colonies were picked out and further cultured in a liquid CDM, and bacteria at the logarithmic growth phase were subsequently inoculated in a liquid CDM with the roseoflavin concentration (10, 50, 100, and 200 mg/L) stepwise increased. Finally, the bacterial culture media with 200 mg/L of roseoflavin were evenly spread onto an MRS agar plate and anaerobically incubated at 37 °C for 24 h. The largest colony of each mutated strain was selected and cultured in a liquid CDM and stored at −80 °C for further analysis.

### 2.7. Identification of LAB Riboflavin Biosynthetic Genes by PCR

Five genes in the riboflavin operon that are necessary for riboflavin biosynthesis, including the *ribA*, *ribB*, *ribC*, *ribH*, and *ribG* genes, were checked by PCR. The *Lactobacillus plantarum* strain JDM1 (NCBI Reference Sequence: NC_012984.1) with the complete riboflavin operon was selected for primer design. Primers (Table 1) were designed by using Primer3web (version 4.1.0, http://primer3.ut.ee/), and their specificity was assessed in silico by using OligoCalc (Kibbe WA, Russian, http://biotools.nubic.northwestern.edu/OligoCalc.html). PCR products were finally checked by DNA electrophoresis.

### 2.8. DNA Sequencing Analysis of Riboflavin Operon Upstream Regulatory Region

RYG-YYG-9049 and its No. 10 mutant with the highest riboflavin productivity were selected to check their riboflavin operon upstream regulatory region via PCR and subsequent DNA sequencing. *L. plantarum* NCDO1752 (Genbank: DQ645592) was selected for primer design. Primers were designed by using the Primer3web (version 4.1.0, http://primer3.ut.ee/), and the sense and antisense sequences of primers were 5′-GATAGTAAGCAATCGTGGTA-3′ and 5′-CGTCTTTGACTAATACCGCAC-3′, respectively. The amplified PCR products were purified and sent to Beyotime Biotechnology Company (Shanghai, China) for DNA sequencing, and the data were analyzed by using the DNAMAN software (version 6.0, Lynnon Biosoft, San Ramon, CA, USA). DNA sequencing results were aligned by using NCBI BLAST (https://www.ncbi.nlm.nih.gov/).

### 2.9. Preparation of Soymilk and Soymilk Fermentation

Soymilk was prepared with the method previously described by Gan [26], with minor modifications. Soybeans were washed three times with deionized water and then soaked in deionized water at a ratio of 1:10 (*w*/*v*) for 16 h at room temperature. After the removal of water, the soybeans were homogenized with water (1:9, *w*/*v*) in a domestic grinder (Philips, Amsterdam, The Netherlands). The mixture was then filtered through a 125 μm sieve, and the filtrate was collected and kept at 4 °C for 2 h to precipitate starch. Subsequently, the supernatant was heated consistently at 100 °C for 30 min by using a heating magnetic stirrer, and then it was cooled down to room temperature and aseptically stored at 4 °C for further use.

Before fermentation experiments, RYG-YYG-9049 and its No. 10 mutant were activated in liquid MRS media at 37 °C for 24 h, and the activated bacteria were then recovered as pellets by centrifugation at 9500× *g* for 30 s at room temperature. After washing with a 0.85% NaCl solution twice, the bacterial pellet was resuspended in a small volume of the 0.85% NaCl solution and then diluted to OD_600_ = 0.2. This bacterial suspension was further used for the fermentation experiments. Subsequently, 5 mL of the heated soymilk was inoculated with 4% (*v*/*v*) of the bacterial suspension and then fermented at different temperatures (23, 30, 37, 44, and 50 °C) and for different times (4, 8, 12, 16, 20, and 24 h). For each fermentation experiment, 5 mL of heated aseptic soymilk without bacterial inoculation was used as a control. After fermentation, the pH of the fermented soymilk was immediately measured with an electronic pH meter (Accument AB150, Cole-Parmer, Vernon Hills, IL, USA). Subsequently, 500 μL of the fermented soymilk was diluted with 500 μL of a 1% (*v*/*v*) acetic acid aqueous solution. The mixture was vortexed, heated (95 °C, 6 min), and centrifuged (21,400× *g*, 30 min, 4 °C), and the supernatant was collected and stored at −20 °C for measuring riboflavin content by HPLC.

### 2.10. Statistical Analysis

All the measurements were performed in triplicate—except for the screening of riboflavin-producing LAB, which was performed in duplicate—and expressed as mean ± standard deviation (SD). Statistical analysis was performed with Microsoft Excel 2016 (Redmond, WA, USA) and SPSS 22.0 (IBM SPSS Statistics, IBM, Somers, NY, USA). Multiple comparisons were carried out by one-way analysis of variance (ANOVA) plus a post hoc Tukey test. *p* < 0.05 was defined as statistical significance.

## 3. Results and Discussion

### 3.1. Riboflavin Content Produced by the LAB Isolated from Pickle Juices

Pickles are a common fermented food that is rich in B group vitamins [27]. Various LAB have been found in pickles, such as *L. plantarum*, *L. fermentum*, *L. sakei*, and *Leuconostoc* (*Lc*.) *mesenteroides* [28]. Therefore, 11 commercial pickles made of different foods, including five cabbage, two garlic, one ginger, one bean, one serpent melon, and one bamboo shoot, were chosen as the raw materials in this study for the screening of riboflavin-producing LAB.

**Figure 2:**
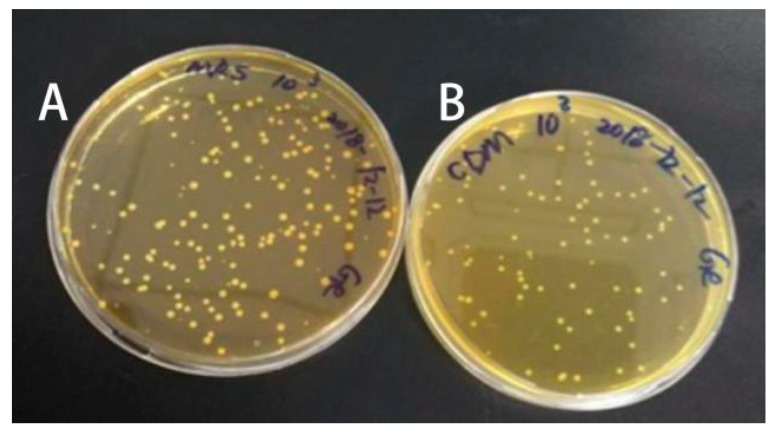
Bacterial colonies obtained upon incubation of the diluted mixture of 11 different pickle juices on MRS plate (**A**) and chemically defined medium (CDM) plate (**B**).

The mixture of pickle juices was initially cultured in MRS and CDM agar plates. Unsurprisingly, the number of LAB colonies grown on the MRS agar plates was 60% more than that on the CDM agar plates (Figure 2). In fact, riboflavin, an essential nutrient for the growth of most LAB strains, is contained in MRS agar plates [29]. Additionally, a CDM, which lacks riboflavin, is commonly used to screen LAB strains that are capable of biosynthesizing riboflavin for colony growth, as previous studies have reported [12,19]. Then, 90 bacterial colonies of wild-type LAB were randomly picked out from the CDM agar plate, cultured, and stabilized for riboflavin production. As shown in Table S1, the content of riboflavin produced by these 90 LAB colonies ranged from 0.096 to 0.734 mg/L, with a 7.6-fold difference. In addition, 52 colonies produced >0.3 mg/L riboflavin, and 27 colonies showed a higher yield of riboflavin (>0.5 mg/L), suggesting that riboflavin-producing capability varies in different LAB colonies.

Three strains, including No. 30, 34, and 49, were found to have the highest riboflavin production, with contents of 0.703, 0.732, and 0.734 mg/L, respectively (Table S1), which were about 2.2–2.8 times higher than that of the *L. plantarum CRL 725* strain (0.26 mg/L) screened from 179 original LAB strains [19]. Additionally, our results were much higher than the riboflavin contents of the *L. plantarum* UNIFG1 and *L. plantarum* UNIFG2 strains that were screened from three natural sourdoughs, both of which has less riboflavin than 0.1 mg/L [29]. Nevertheless, a strain of *L. fermentum* MTCC 8711, which had similar content of riboflavin to that of No. 49, which was screened from 37 homemade fermented milk samples and was reported to produce 2.29 mg/L riboflavin when using the CDM [30]. It seems that different raw materials vary in biochemical composition, which might have an effect on the growth and metabolism of different LAB strains, resulting in diverse riboflavin production [31,32]. However, fine LAB colonies showed a non-detectable riboflavin content (Table S1), probably due to the decomposition or utilization of riboflavin by certain LAB, such as *Lactococcus lactis* subsp. *cremoris* NZ9000, *L. plantarum* NCDO1752, and *Lc. mesenteroides* NCDO2028, in which riboflavin can be used as a nutrient for colony growth, resulting in very low or even no accumulation of riboflavin [20,25].

### 3.2. Identification of LAB Strains with High Riboflavin-Producing Capability

The species identification of LAB was based on 16S rDNA sequencing analysis that was combined with morphological analysis. Three LAB strains (No. 30, 34, and 49) that had the highest yield of riboflavin were purified, activated, and inoculated on an MRS agar plate for 24 h. The colony morphology of the three LAB strains was white, round, smooth, and with a tidy edge as well as bulge and smooth surface. Gram staining and a catalase test showed that the three strains were Gram-positive and catalase-negative.

The 16S rDNA of the three strains was sequenced, analyzed, and compared with the corresponding sequences of known strains registered in the BLAST gene database for homology alignment. According to the results, all three riboflavin high-yield strains were identified as *L. plantarum*, with a similarity of 99.86–99.93%. Thus, the wild LAB strain No. 49, which produced the highest riboflavin content among the three strains, was named *L. plantarum* RYG-YYG-9049 and selected for spontaneous mutations to further enhance riboflavin production.

### 3.3. Riboflavin Production Was Increased in Roseoflavin-Induced Mutant Strains of RYG-YYG-9049

**Figure 3:**
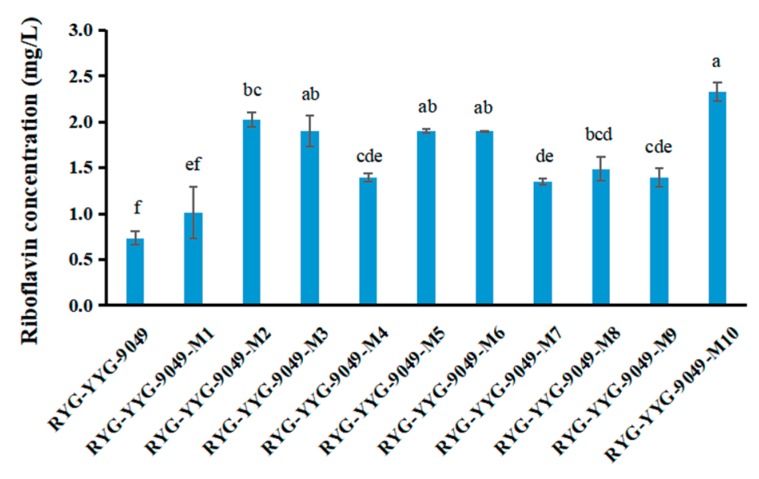
The concentration of riboflavin produced by the RYG-YYG-9049 strain and its ten mutant strains. Values with different lowercase letters indicate statistical significance (*p* < 0.05).

Roseoflavin, a toxic riboflavin analog, is often used as an efficient chemical mutagen to induce bacterial spontaneous mutation that is accompanied with increased riboflavin production [33]. In this study, roseoflavin was used to induce the spontaneous mutation of the RYG-YYG-9049 strain, and 10 mutant strains were then randomly selected in CDM agar plates. Compared with the wild-type RYG-YYG-9049 strain, all 10 mutant strains could produce more riboflavin (Figure 3), suggesting that riboflavin-biosynthesis capability was enhanced in the mutant strains. Particularly, the mutant strain No. 10 (noted as RYG-YYG-9049-M10) showed the highest riboflavin content (2.332 mg/L), which was three times higher than that of its wild-type strain (0.734 mg/L). In addition, the RYG-YYG-9049-M10 strain in our study also produced a much higher riboflavin content than the roseoflavin-mutated LAB strains in previous studies, such as the *Lc. mesenteroides* NCDO2028 mutant CB207 strain (0.5 mg/L) [25], the *L. plantarum* CRL 725 variant (G) strain (1.1 mg/L) [19], and the mutant *L. fermentum* PBCC11.5 strain (1.203 mg/L) [12]. Therefore, the RYG-YYG-9049-M10 strain was a riboflavin high-producing LAB.

### 3.4. Roseoflavin-Induced Mutation Did Not Change Riboflavin Biosynthetic Genes in RYG-YYG-9049-M10

It is known that LAB cannot produce riboflavin when lacking any of the *ribA*, *ribB*, *ribG*, or *ribH* genes [25,34]. The results of PCR analysis indicated the presence of the genes that encode enzymes involved in riboflavin biosynthesis (*ribABCGH*) in both the wild-type RYG-YYG-9049 (Figure S1A) and its mutant RYG-YYG-9049-M10 strains (Figure S1B). Additionally, the DNA sequence of the fine *rib* genes in those two strains showed a very high similarity, ranging from 98.25% to 99.9%, suggesting that roseoflavin did not change the *rib* genes. In addition, previous studies have reported that the upstream regulatory sequence of the *rib* operon in *Lc. mesenteroides* NCDO2028 and *L. plantarum* NCDO1752 were modified after roseoflavin treatment [25]. Therefore, the roseoflavin-induced mutation sites of LAB are more likely to exist in the upstream regulatory region of the *rib* operon.

### 3.5. Roseoflavin Induced the Mutation of the Upstream Regulatory Region of Rib Operon in RYG-YYG-9049-M10

In order to investigate whether roseoflavin can induce the mutation of the upstream regulatory region of the *rib* operon, a DNA sequencing analysis was performed to compare the DNA sequence of the upstream regulatory region of the *rib* operon in wild-type RYG-YYG-9049 and RYG-YYG-9049-M10.

**Figure 4:**
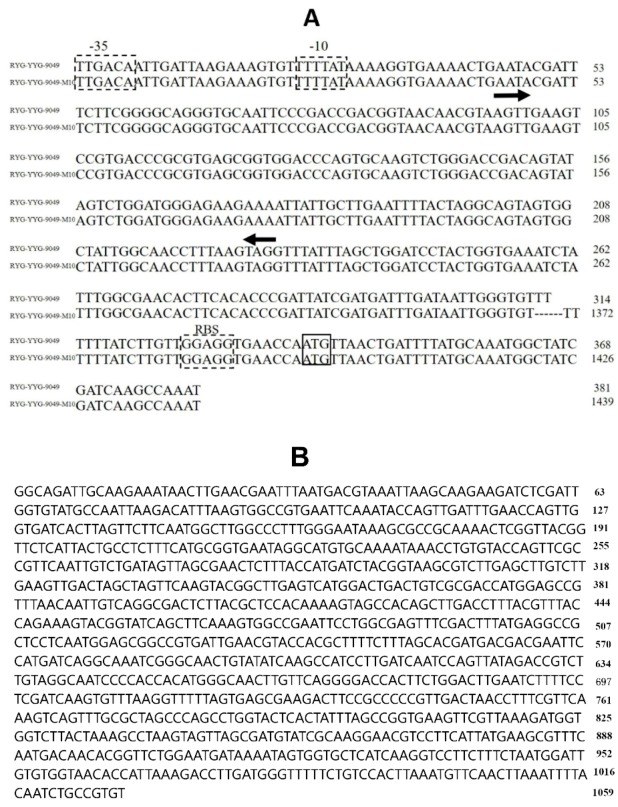
(**A**) Alignment of the rib operon regulatory region of RYG-YYG-9049 and its mutant strain. The predicted −10 and −35 recognition sequences and ribosomal binding sites (RBS) are boxed. The ellipsis represents the increased gene sequence of the mutant strain. The *ribG* start codon is boxed with solid lines. The RFN element is indicated by the arrows below. (**B**) The inserted 1059-bp gene sequence.

Compared with the wild-type strain, RYG-YYG-9049-M10 showed an inserted DNA fragment located in the upstream regulatory region of the *rib* operon, which is represented by ellipsis in Figure 4A. In fact, this 1059-bp DNA fragment (Figure 4B) was inserted in an untranslated region between the RFN-encoding DNA (RFN element conserves a mononucleoside riboswitch that exists in 5′ untranslated regions of prokaryotic mRNA, which is responsible for the biosynthesis of FMN and the coding of transporters) and the ribosomal binding site of the *rib* operon. Apart from this change, other DNA sequences of the upstream regulatory region of the *rib* operon were identical between RYG-YYG-9049 and RYG-YYG-9049-M10. Therefore, this insertion was the most likely cause of the observed riboflavin increase in RYG-YYG-9049-M10.

DNA mutation has been reported in the *Lactococcus lactis* subsp. *cremoris* NZ9000 mutated strain, though mainly with some changes of single bases and partial sequence deletion, which has been presumed to enhance the transcription of the *rib* operon [20]. In a later study, Burgess et al. found that all the mutation sites of the *L. plantarum* NCDO1752 mutant were located in the region encompassing part of the RFN-encoding sequence, with some modifications of single bases and partial sequence loss [25]. It has been suggested that the position of mutations might affect the stability of the terminator structure, leading to a less favorable formation of this terminator, thereby allowing the *rib* operon to be continuously transcripted [25]. Gene deletion and/or single-base modification in the upstream regulatory region of the *rib* operon are common types of roseoflavin-induced spontaneous mutation [25,34,35]. In the present study, it was interesting to find a novel mutation type with an inserted gene fragment in the untranslated region of the upstream regulatory region that could be considered as a new mechanism of the roseoflavin-induced increase of riboflavin in LAB.

In order to confirm the riboflavin-producing stability of this mutant strain, 60 generations of RYG-YYG-9049-M10 were continuously cultured. The results showed that there was no significant change in the riboflavin yield of the strain after 60 generations of culture (data not shown). This indicated that the mutant was stable after roseoflavin-induced mutation, consistent with previous studies [19,20,25], and this stable strain is of great interest to be applied in the food industry to develop LAB-fermented foods that are rich in riboflavin.

### 3.6. RYG-YYG-9049 and Its M10 Fermentation Increased Riboflavin Content in Soymilk

**Figure 5:**
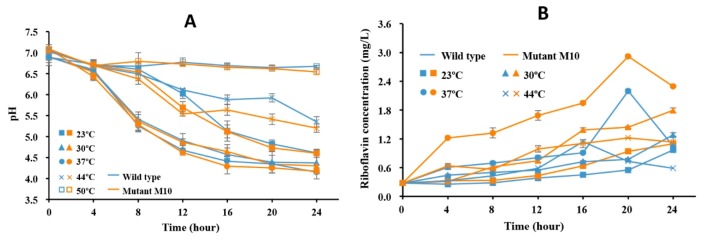
(**A**) pH value of fermented soymilk by the RYG-YYG-9049 wild strain and its No. 10 mutant strain under different conditions. (**B**) Changes of riboflavin value in soymilk that was inoculated with the bacteria at different time and temperatures.

The wild-type RYG-YYG-9049 and its M10 strains were applied to ferment soymilk to increase its riboflavin content. The effects of main fermentation parameters, including temperature (23, 30, 37, 44, and 50 °C) and time (4, 8, 12, 16, 20, and 24 h) were investigated on riboflavin production. A decrease in the pH of the fermented soymilk was observed during the fermentation process (Figure 5A), but this decrease was very slight at a fermentation temperature of 50 °C because of no accumulation of lactic acid, indicating that the strain could not grow or even died at 50 °C. Therefore, riboflavin in soymilk fermented at 50 °C was detected. As shown in Figure 5B, the highest riboflavin yields were achieved by fermentation at 37 °C for 20 h in both RYG-YYG-9049 and its M10 strain fermented soymilk. Under this optimal fermentation condition, the riboflavin content reached 2.920 ± 0.008 mg/L in the soymilk that was fermented by RYG-YYG-9049-M10, about 10-fold higher than that in soymilk without fermentation (0.273 mg/L). A similar result was reported by Juarez del Valle et al.; in their study, a six-fold increase in riboflavin content was obtained when the soymilk was fermented by using a mutant *L. plantarum* CRL725 [19]. Thus, the application of riboflavin-overproducing LAB could be an effective strategy to enhance riboflavin production in fermented soymilk, as well as in other fermented food products. For example, the fermentation of bread and pizza with *L. plantarum* UNIFG1 and *L. plantarum* UNIFG2 has been shown to lead to an increase in riboflavin content in the range of three-to-four times [23]. In addition, the inoculation of the *L. fermentum* PBCC11 mutant strain in dough resulted in a two-to-three-fold increase of riboflavin content in sourdough after a 16-h fermentation [12].

According to the European Food Information Council (EFIC, 2006), the recommended daily allowance (RDA) of riboflavin at 1.4 mg/day can normally be supplied to an adult by a balanced diet [36]. On the basis of our result, 100 mL of soymilk that was fermented by the RYG-YYG-9049-M10 strain could provide 20.9% of the RDA, while that which was fermented with the wild-type RYG-YYG-9049 strain could provide 15.7% of the RDA. Both fermented milks were significantly better than the unfermented soymilk, which could only provide 1.95% of the RDA. Therefore, the use of the RYG-YYG-9049-M10 strain can also be considered as a biotechnological strategy to obtain riboflavin-enriched soymilk. However, for a strain destined for industrial food production, its survival and functionality under in vitro and in vivo conditions should be tested in the future in order to evaluate whether it can be considered as a probiotic strain for food production [37]. In addition, the adaptability of the strain during food production and storage also needs to be considered, because food stresses, such as pH, freezing, temperature, and osmotic pressure, may have a significant influence on its performance [36]. Though the temperature was tested in the present study, the influences of other factors still need to be clarified in the future. Finally, the combination of this strain with commercial strains may be a good strategy to improve the nutritional value and sensorial quality of the products.

## 4. Conclusions

Wild-type riboflavin-producing LAB were successfully isolated from a mixture of pickle juices, and the strain with the highest riboflavin production was identified and denominated as RYG-YYG-9049. The further roseoflavin mutagenesis of this strain generated 10 roseoflavin-resistant mutants, among which the No. 10 mutant (denominated as RYG-YYG-9049-M10) produced the highest riboflavin content. According to comparative sequencing analysis, a new mutation in the upstream regulatory region of the *rib* operon in this mutant was observed, and this was the most likely cause for the increase in riboflavin production. Finally, this mutant strain was applied to ferment raw soymilk in order to produce a novel riboflavin-fortified soymilk with optimized fermentation parameters. The RYG-YYG-9049-M10 strain could increase riboflavin content by 10 times in fermented soymilk in a 20-h fermentation at 37 °C, and the fermented soymilk that was rich in riboflavin could be considered as a good dietary source of riboflavin to prevent or reduce the incidence of riboflavin-deficiency related diseases. The concept of the in situ production of riboflavin with carefully selected LAB can open a way to develop novel food products that are destined for different, specific groups of people, such as the elderly, children, pregnant women, sportsmen, vegetarians, and adolescents.

## Figures and Tables

**Table 1:** Primer sequences used for PCR analysis.

Gene	Primer
*ribA*	f-5′-GAGCGCCGGTATGATATTGC-3′
	r-5′-GTGTCCCTCCGTTAGTGTGA-3′
*ribB*	f-5′-TGGCAGTTGATGGGGTTAGT-3′
	r-5′-CTAATTGCCGGGCCAAGTAC-3′
*ribC*	f-5′-CGCTACAGGTGGACCGACTA-3′
	r-5′-GCATTCAACCCGACAAGGTA-3′
*ribG*	f-5′-ACGTACCAAAATCCCCAGGT-3′
	r-5′-ACATCCACCTCAGCATGGTC-3′
*ribH*	f-5′-ACCGGGAGCTTTTGAGATTC-3′
	r-5′-ATAGTCAAAGTGCGCGGTTG-3′

## Supplementary Materials

The following are available online at https://www.mdpi.com/2304-8158/9/1/88/s1, Table S1: The content of riboflavin produced by 90 LAB colonies obtained from CDM plate. Figure S1: (A) PCR products of the riboflavin biosynthetic genes of RYG-YYG-9049 strain. (B) PCR products of the riboflavin biosynthetic genes of RYG-YYG-9049-M10 strain.
