# Supplementary material for: Screening and Spontaneous Mutation of Pickle-Derived Lactobacillus plantarum with Overproduction of Riboflavin, Related Mechanism, and Food Application
Source: Foods. 2020 Jan 14;9(1):88. doi: 10.3390/foods9010088 (PMC7022482; doi:10.3390/foods9010088)
Supplement: Supplementary file 1 [file foods-09-00088-s001.zip › foods-654720-SI.docx]

**Supplementary Materials:**

**Table S1:** The content of riboflavin produced by 90 LAB colonies obtained from CDM plate.

| **No.** | **Riboflavin (mg/L)** | **No.** | **Riboflavin (mg/L)** | **No.** | **Riboflavin (mg/L)** |
| --- | --- | --- | --- | --- | --- |
| 1 | 0.096 ± 0.087 | 31 | 0.484 ± 0.174 | 61 | 0.589 ± 0.006 |
| 2 | 0.175 ± 0.076 | 32 | 0.384 ± 0.097 | 62 | 0.551 ± 0.038 |
| 3 | 0.199 ± 0.127 | 33 | 0.344 ± 0.064 | 63 | 0.425 ± 0.167 |
| 4 | 0.188 ± 0.072 | **34** | **0.732 ± 0.021** | 64 | 0.510 ± 0.209 |
| 5 | 0.114 ± 0.013 | 35 | 0.405 ± 0.082 | 65 | 0.504 ± 0.092 |
| 6 | 0.144 ± 0.024 | 36 | 0.387 ± 0.065 | 66 | 0.184 ± 0.160 |
| 7 | 0.276 ± 0.138 | 37 | 0.324 ± 0.099 | 67 | 0.460 ± 0.073 |
| 8 | 0.169 ± 0.007 | 38 | 0.255 ± 0.015 | 68 | 0.532 ± 0.077 |
| 9 | 0.177 ± 0.016 | 39 | 0.358 ± 0.153 | 69 | 0.484 ± 0.017 |
| 10 | 0.273 ± 0.095 | 40 | 0.546 ± 0.013 | 70 | 0.313 ± 0.038 |
| 11 | 0.295 ± 0.152 | 41 | 0.300 ± 0.047 | 71 | ND ^1^ |
| 12 | 0.312 ± 0.096 | 42 | 0.360 ± 0.031 | 72 | 0.368 ± 0.029 |
| 13 | 0.226 ± 0.062 | 43 | 0.367 ± 0.011 | 73 | 0.545 ± 0.071 |
| 14 | 0.252 ± 0.017 | 44 | 0.577 ± 0.009 | 74 | 0.588 ± 0.032 |
| 15 | 0.290 ± 0.015 | 45 | 0.509 ± 0.032 | 75 | 0.038 ± 0.036 |
| 16 | 0.264 ± 0.156 | 46 | 0.519 ± 0.037 | 76 | 0.100 ± 0.028 |
| 17 | 0.581 ± 0.027 | 47 | 0.663 ± 0.053 | 77 | 0.158 ± 0.008 |
| 18 | 0.315 ± 0.085 | 48 | 0.535 ± 0.067 | 78 | 0.195 ± 0.146 |
| 19 | 0.325 ± 0.132 | **49** | **0.734 ± 0.073** | **79** | 0.200 ± 0.138 |
| 20 | 0.305 ± 0.100 | 50 | 0.510 ± 0.026 | 80 | ND ^1^ |
| 21 | 0.444 ± 0.051 | 51 | ND ^1^ | 81 | 0.470 ± 0.160 |
| 22 | 0.481 ± 0.106 | 52 | ND ^1^ | 82 | 0.436 ± 0.124 |
| 23 | 0.310 ± 0.070 | 53 | 0.638 ± 0.020 | 83 | 0.329 ± 0.130 |
| 24 | 0.658 ± 0.060 | 54 | 0.575 ± 0.062 | 84 | 0.291 ± 0.016 |
| 25 | 0.225 ± 0.203 | 55 | 0.602 ± 0.078 | 85 | 0.221 ± 0.184 |
| 26 | 0.189 ± 0.157 | 56 | 0.690 ± 0.025 | 86 | 0.186 ± 0.012 |
| 27 | 0.266 ± 0.155 | 57 | 0.585 ± 0.112 | 87 | 0.118 ± 0.069 |
| 28 | 0.189 ± 0.193 | 58 | 0.579 ± 0.110 | 88 | 0.164 ± 0.084 |
| 29 | 0.297 ± 0.073 | 59 | 0.640 ± 0.022 | 89 | 0.165 ± 0.066 |
| **30** | **0.703 ± 0.020** | 60 | 0.589 ± 0.061 | 90 | ND ^1^ |

Data were shown as mean ± standard deviation. ^1^ Not Detected.


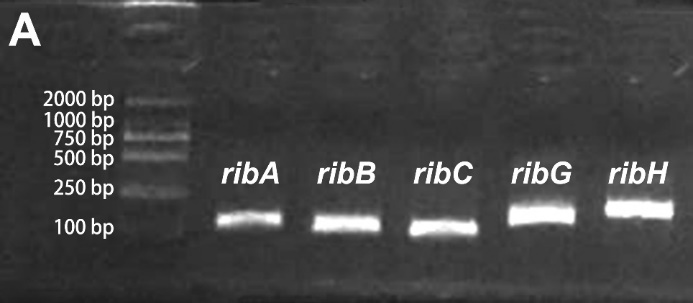


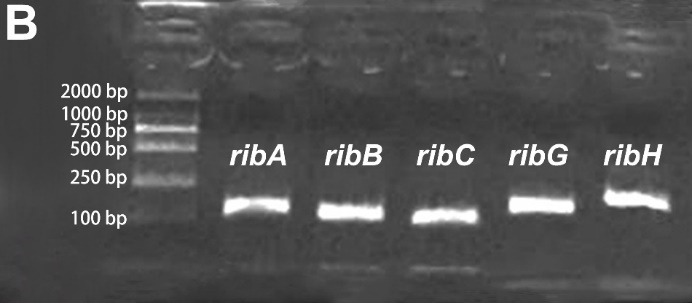


**Figure S1:** (A) PCR products of the riboflavin biosynthetic genes of RYG-YYG-9049 strain. (B) PCR products of the riboflavin biosynthetic genes of RYG-YYG-9049-M10 strain.
